# Suicide attempts in U.S. Army combat arms, special forces and combat medics

**DOI:** 10.1186/s12888-017-1350-y

**Published:** 2017-05-25

**Authors:** Robert J. Ursano, Ronald C. Kessler, James A. Naifeh, Holly Herberman Mash, Carol S. Fullerton, Tsz Hin Hinz Ng, Pablo A. Aliaga, Gary H. Wynn, Hieu M. Dinh, James E. McCarroll, Nancy A. Sampson, Tzu-Cheg Kao, Michael Schoenbaum, Steven G. Heeringa, Murray B. Stein, on behalf of the Army STARRS collaborators, James Wagner, Kenneth Cox, David M. Benedek, Laura Campbell-Sills, Nancy Gebler, Robert K. Gifford, Sonia Jain, Lisa Lewandowski-Romps, Paul E. Hurwitz, Matthew K. Nock, Nancy A. Sampson, Patcho Santiago, Alan M. Zaslavsky

**Affiliations:** 1 Center for the Study of Traumatic Stress, Department of Psychiatry, Uniformed Services University of the Health Sciences, 4301 Jones Bridge Road, Bethesda, MD 20814 USA; 2 Department of Health Care Policy, Harvard Medical School, 180 Longwood Avenue, Boston, MA 02115 USA; 3 Department of Preventive Medicine and Biostatistics, Uniformed Services University of the Health Sciences, 4301 Jones Bridge Road, Bethesda, MD 20814 USA; 4 Office of Clinical and Population Epidemiology Research, Division of Services and Intervention Research, National Institute of Mental Health, 6001 Executive Blvd, Room 7137, MSC 9635, Bethesda, MD 20892 USA; 5 Institute for Social Research, University of Michigan, 426 Thompson Street, Ann Arbor, MI 48106-1248 USA; 6 Departments of Psychiatry and Family Medicine & Public Health, University of California San Diego, 8939 Villa La Jolla Drive, Suite 200, La Jolla, California, 92037 USA; 7 VA San Diego Healthcare System, 8810 Rio San Diego Drive, San Diego, CA 92108 USA

**Keywords:** Suicide attempt, Military, Occupation

## Abstract

**Background:**

The U.S. Army suicide attempt rate increased sharply during the wars in Iraq and Afghanistan. Risk may vary according to occupation, which significantly influences the stressors that soldiers experience.

**Methods:**

Using administrative data from the Army Study to Assess Risk and Resilience in Servicemembers (Army STARRS), we identified person-month records for all active duty Regular Army enlisted soldiers who had a medically documented suicide attempt from 2004 through 2009 (*n* = 9650) and an equal-probability sample of control person-months (*n* = 153,528). Logistic regression analyses examined the association of combat occupation (combat arms [CA], special forces [SF], combat medic [CM]) with suicide attempt, adjusting for socio-demographics, service-related characteristics, and prior mental health diagnosis.

**Results:**

In adjusted models, the odds of attempting suicide were higher in CA (OR = 1.2 [95% CI: 1.1–1.2]) and CM (OR = 1.4 [95% CI: 1.3–1.5]), but lower in SF (OR = 0.3 [95% CI: 0.2–0.5]) compared to all other occupations. CA and CM had higher odds of suicide attempt than other occupations if never deployed (ORs = 1.1–1.5) or previously deployed (ORs = 1.2–1.3), but not when currently deployed. Occupation was associated with suicide attempt in the first ten years of service, but not beyond. In the first year of service, primarily a time of training, CM had higher odds of suicide attempt than both CA (OR = 1.4 [95% CI: 1.2–1.6]) and other occupations (OR = 1.5 [95% CI: 1.3–1.7]). Discrete-time hazard functions revealed that these occupations had distinct patterns of monthly risk during the first year of service.

**Conclusions:**

Military occupation can inform the understanding suicide attempt risk among soldiers.

**Electronic supplementary material:**

The online version of this article (doi:10.1186/s12888-017-1350-y) contains supplementary material, which is available to authorized users.

## Background

 Suicidal behavior among U.S. Army soldiers increased substantially during the wars in Iraq and Afghanistan, [1, 2] with the rates of suicide death more than doubling from 2001 (9/100,000) through 2009 (22/100,000) and surpassing the adjusted civilian rate in 2008 [3]. Although the Army has implemented a variety of screening [4–6] and prevention programs, [7] identifying soldiers at risk of suicide remains a significant challenge. Research on military suicide has often emphasized the importance of deployment history, with mixed results [1, 3, 8–11]. Deployment experiences vary substantially depending on a soldier’s military occupation. A meta-analysis found that suicidal outcomes were more strongly associated with particular combat experiences (e.g., killing, exposure to death) than with deployment in general, [12] suggesting that occupations characterized by direct combat exposure may have a higher suicide risk than other occupations [13, 14].

Soldiers with a combat arms (CA) occupation (e.g., infantry, airborne) have the highest likelihood of combat exposure, including frequent contact with enemy forces and increased risk of death and injury. CA soldiers tend to be at high risk for posttraumatic stress reactions, suicidality, and other mental health problems relative to other military occupations [15–17]. Special forces (SF) are elite, highly trained soldiers who engage in frequent, often unconventional warfare operations. Although SF is a branch of CA, it warrants distinct consideration. Soldiers who successfully complete the rigorous selection process and training for SF may have unique characteristics [18–20] that make them more resilient than other soldiers [21]. Combat medics (CM) are also of particular interest, as they serve dual roles as both soldiers and healthcare providers [22]. CM can experience direct combat exposure while embedded with infantry units [23, 24] and are also directly exposed to the severe injury and death of soldiers they attempt to save.

The relevance of occupation extends well beyond exposure to combat-related stressors. The content, duration, and stressors associated with training vary considerably by occupation. The first year of service is largely a time of training and carries particularly high risk for suicide attempts [25, 26]. CM have intense performance demands during this time, including 16 weeks of Advanced Individual Training in which they must achieve proficiency equal to or greater than an emergency medical technician. The training demands on CA soldiers, while also intense, are very different in nature.

We examine the association of occupation with suicide attempts among enlisted soldiers in U.S. Army. Enlisted soldiers accounted for nearly 99% of suicide attempts from 2004 through 2009 [26]. Using administrative data from the Army Study to Assess Risk and Resilience in Servicemembers (Army STARRS), [27] we focus on CA, SF, and CM, three occupations with high likelihood of direct combat exposure. CA soldiers are traditionally a population of intense focus in military mental health research, however, less is known about the health and functioning of SF and CM soldiers. We also examined whether the association of occupation with suicide attempt varied by deployment status and time in service [28, 29].

## Methods

### Sample

This longitudinal, retrospective cohort study used data from the Army STARRS Historical Administrative Data Study (HADS), which integrates 38 Army and Department of Defense administrative data systems. The HADS includes deidentified individual-level person-month records for all soldiers on active duty between January 1, 2004 and December 31, 2009 (*n* = 1.66 million) [30]. This component of Army STARRS was approved by the Institutional Review Boards of the Uniformed Services University of the Health Sciences, University of Michigan Institute for Social Research, University of California, San Diego, and Harvard Medical School.

The analytic sample for the current investigation included all 9650 Regular Army enlisted soldiers who attempted suicide from 2004 through 2009 (excluding officers and activated Army National Guard and Army Reserve), plus an equal-probability sample of 153,523 control person-months. Data were analyzed using a discrete-time survival framework with person-month as the unit of analysis, [31] such that each month in the career of a soldier was treated as a separate observational record. Given that discrete-time survival coefficients can be estimated without bias when control person-months are randomly subsampled and weighted using the logic of case–control analysis, [32] we reduced computational intensity by selecting from the population an equal-probability 1:200 sample of control person-months stratified by gender, rank, time in service, deployment status (never, currently, or previously deployed), and historical time. Control person-months excluded all soldiers with a documented suicide attempt or other non-fatal suicidal event (e.g., suicide ideation) [2] and person-months in which a soldier died. Each control person-month was assigned a weight of 200 to adjust for under-sampling.

### Measures

#### Suicide attempt

Soldiers who attempted suicide were identified using administrative records from: the Department of Defense Suicide Event Report (DoDSER), [33] a DoD-wide surveillance mechanism that aggregates information on suicidal behaviors via a standardized form completed by medical providers at DoD treatment facilities; and ICD-9-CM diagnostic codes E950-E958 (indicating self-inflicted poisoning or injury with suicidal intent) from the Military Health System Data Repository (MDR), Theater Medical Data Store (TMDS), and TRANSCOM (Transportation Command) Regulating and Command and Control Evacuating System (TRAC^2^ES), which together provide healthcare encounter information from military and civilian treatment facilities, combat operations, and aeromedical evacuations (see Additional file 1: Table S1). We excluded suicide deaths and DoDSER records indicating only suicide ideation. The E959 code (late effects of a self-inflicted injury) was excluded, as it confounds the temporal relationships between the predictor variables and suicide attempt [34]. Records from different data systems were cross-referenced to ensure all cases represent unique soldiers. For soldiers with multiple suicide attempts, we selected the first attempt using a hierarchical classification scheme that prioritized DoDSER records due to that system’s more extensive reporting requirements [2].

#### Military occupation

Occupational information for each person-month was obtained from the Defense Manpower Data Center. The current study focused on four occupational categories: combat arms (CA), special forces (SF), combat medics (CM), and all other (AO) occupations (see Additional file 1: Table S2). Functional roles and duties of each occupation were examined to facilitate classification. CA included occupations that were identified, based on expert consensus, as those most typically exposed to direct combat. This includes some, but not all, of the occupations traditionally classified as combat arms [35, 36].

#### Other predictors

Socio-demographic (gender, current age, race, education, marital status), service-related (age at Army entry, time in service [≤ 1 year, 2 years, 3–4 years, 5–10 years, >10 years], deployment status [never, currently, or previously deployed]) and previous mental health diagnosis variables were also drawn from Army/DoD administrative data systems (see Additional file 1: Table S1). The indicator variable for previous mental health diagnosis during Army service combined categories derived from ICD-9-CM codes (e.g., major depression, bipolar disorder, posttraumatic stress disorder, personality disorders), excluding postconcussion syndrome, tobacco use disorder, and supplemental V-codes that are not disorders (e.g., stressors/adversities, marital problems) when those were the only recorded mental health diagnoses (see Additional file 1: Table S3).

### Analysis

All analyses were conducted using SAS version 9.3 [37]. We first examined the multivariate association of the four occupational categories with suicide attempt in logistic regression analyses that adjusted for socio-demographics and service-related variables. We then conducted a sensitivity analysis to examine the robustness of occupation as a predictor by adding to the model an indicator for any previous mental health diagnosis. Adjusting for socio-demographic and service-related variables, we then separately examined the interaction of occupation with deployment status (never, currently, or previously deployed) and time in service. Significant interactions were examined more closely through stratification. Logistic regression coefficients were exponentiated to obtain odds-ratios (OR) and 95% confidence intervals (CI). Final model coefficients were used to generate a *standardized* risk estimate [38] (SRE; number of suicide attempters per 100,000 person-years) for each category of each predictor under the model assuming other predictors were at their sample-wide means. All logistic regression models included a dummy predictor for calendar month and year to control for increasing rates of suicide attempt from 2004 through 2009 [2]. Coefficients of other predictors can consequently be interpreted as averaged within-month associations based on the assumption that effects of other predictors do not vary over time.

We used discrete-time hazard functions and linear spline models to examine risk of suicide attempt as a function of time and occupation. Separate analyses for each occupational category estimated risk by months since entering service among soldiers in their first year. Splines (piecewise linear functions) were examined using chi-square difference tests, deviance, and the Akaike Information Criterion to test whether knots and additional linear segments improved model fit to assess nonlinearities in changes in risk by time in service.

## Results

### Risk of suicide attempt by occupation

Nearly one-quarter of enlisted soldiers were CA (23.3%), 1.2% were SF, and 4.8% were CM, with 70.7% comprising AO. Out of 9650 total enlisted suicide attempters, 26.0% (*n* = 2506) were CA, 0.1% (*n* = 16) were SF, 7.1% (*n* = 682) were CM, and 66.8% (*n* = 6446) were AO. Adjusting for socio-demographic and service-related variables (not shown), the overall association of occupation with suicide attempt was significant (χ^2^_3_ = 126.2, *p* < 0.0001). Odds were higher for CA (OR = 1.2 [95% CI: 1.1–1.2]) and CM (OR = 1.4 [95% CI: 1.3–1.5]) compared to AO, whereas SF had lower odds (OR = 0.3 [95% CI: 0.2–0.5]). The SRE was highest for CM (504/100,000 person-years [PY]), followed by CA (417/100,000 PY), and AO (357/100,000 PY). SF had the lowest SRE at 102/100,000 PY (Table 1). Pairwise analyses (not shown) indicated that CM were significantly more likely to attempt suicide than CA (OR = 1.2 [95% CI: 1.1–1.3]), and this remained unchanged when comparing CM to CA among males only (OR = 1.2 [95% CI: 1.1–1.3]), which is important given that female soldiers were not permitted to have a CA or SF occupation at this time.

**Table 1:** Multivariate association of military occupation with suicide attempt among Regular Army enlisted soldiers, adjusting for socio-demographic and service-related variables^a, b^

	OR	(95% CI)	Cases (*n*)	Total (*n*)^c^	Rate^d^	Pop %^e^	SRE^f^
Occupation							
Combat arms	1.2*	(1.1–1.2)	2506	7,159,106	420	23.3	417
Special forces	0.3*	(0.2–0.5)	16	368,016	52	1.2	102
Combat medic	1.4*	(1.3–1.5)	682	1,470,882	556	4.8	504
Other	1.0	–	6446	21,716,246	356	70.7	357
χ^2^_3_	126.2*						

^a^The sample of enlisted soldiers (*n* = 9650 cases, 153,523 control person-months) is a subset of the total sample (*n* = 193,617 person-months) from the Army STARRS Historical Administrative Data Study (HADS). Control person-months were assigned a weight of 200 to adjust for under-sampling

^b^Logistic regression models included gender, age at Army entry, current age, race/ethnicity, education, marital status, time in service (≤ 1 year, 2 years, 3–4 years, 5–10 years, >10 years), deployment status (never, currently, or previously deployed), and military occupation. The model also included a dummy predictor variable for calendar month and year to control for secular trends

^c^Total includes both cases (i.e., soldiers with a suicide attempt) and control person-months

^d^Rate per 100,000 person-years, calculated based on n_1_/n_2_, where n_1_ is the unique number of soldiers within each category and n_2_ is the annual number of person-*years*, not person-*months*, in the population (*n* = 3.08 million)

^e^Pop % = percent of the population of enlisted soldiers

^f^SRE = Standardized risk estimates (suicide attempters per 100,000 person-years) were calculated assuming other predictors were at their sample-wide means

*p < 0.05

We next included an indicator for any prior mental health diagnosis while in the Army (see Additional file 1: Table S4). The association of occupation with suicide attempt remained significant (χ^2^_3_ = 96.2, *p* < 0.0001). The ORs for CA (OR = 1.2 [95% CI: 1.1–1.3]), SF (OR = 0.5 [95% CI: 0.3–0.7]), and CM (OR = 1.3 [95% CI: 1.2–1.4]) changed very little after accounting for prior mental health diagnosis. In a similar analysis among males the results were unchanged: CA (OR = 1.2 [95% CI: 1.2–1.3]), SF (OR = 0.5 [95% CI: 0.3–0.7]), and CM (OR = 1.3 [95% CI: 1.2–1.4]) (see Additional file 1: Table S5). Among females the results for CM were also unchanged (OR = 1.3 [95% CI: 1.2–1.5]). Given that inclusion of prior mental health diagnosis did not alter the influence of occupation on risk of suicide attempt, we did not include mental health diagnosis in subsequent analyses. SF soldiers were excluded from subsequent analyses owing to their small numbers.

In separate analyses that adjusted for socio-demographic and service-related predictors, the two-way interactions of occupation with deployment status (χ^2^_4_ = 43.8, *p* < 0.0001) and time in service (χ^2^_8_ = 47.2, *p* < 0.0001) were significant. Their three-way interaction was non-significant.

We stratified by deployment status and examined occupation in separate models that adjusted for the socio-demographic and service-related covariates. The association of occupation with suicide attempt was significant among never deployed (χ^2^_2_ = 67.2, *p* < 0.0001) and previously deployed (χ^2^_2_ = 32.8, *p* < 0.0001), but not among currently deployed (χ^2^_2_ = 4.5, *p* = 0.10). CA had higher odds of suicide attempt than AO in those never deployed (OR = 1.1 [95% CI: 1.0–1.2]; SRE = 610/100,000 PY) and previously deployed (OR = 1.3 [95% CI: 1.2–1.4]; SRE = 358/100,000 PY). CM also had higher odds than AO in never deployed (OR = 1.5 [95% CI: 1.3–1.6]; SRE = 801/100,000 PY) and previously deployed (OR = 1.2 [95% CI: 1.0–1.5]; SRE = 352/100,000 PY) (Table 2; see Additional file 1: Table S6 for supplemental counts and rates by occupation and deployment status). In pairwise analyses CM had significantly higher odds relative to CA in those never deployed (OR = 1.3 [95% CI: 1.2–1.5]), but not in those previously deployed. Stratifying by occupation, deployment status was associated with suicide attempt among CA (χ^2^_2_ = 367.0, *p* < 0.0001), CM (χ^2^_2_ = 29.8, *p* < 0.0001), and AO (χ^2^_2_ = 378.1, *p* < 0.0001). Among CA, those never deployed (OR = 3.3 [95% CI: 2.8–3.8]) or previously deployed (OR = 3.8 [95% CI: 3.3–4.4]) had higher odds than currently deployed, and previously deployed CA had higher odds than those never deployed (OR = 1.2 [95% CI: 1.0–1.3]). Similarly, among CM, never deployed (OR = 1.9 [95% CI: 1.4–2.6]) or previously deployed (OR = 2.4 [95% CI: 1.7–3.2]) had higher odds of suicide attempt than those currently deployed. However, there was no difference between CM who were never deployed and previously deployed. Among AO, never deployed (OR = 2.1 [95% CI: 1.9–2.3]) and previously deployed (OR = 2.5 [95% CI: 2.3–2.8]) had higher odds than those currently deployed, and those previously deployed had higher odds than those never deployed (OR = 1.2 [95% CI: 1.1–1.3]) (see Additional file 1: Table S7a and S7b for pairwise analyses by occupation and deployment status).

**Table 2:** Multivariate associations of military occupation with suicide attempt among Regular Army enlisted soldiers stratified by deployment status^a, b^

	Deployment Status								
	Never Deployed (*n* = 67,336)			Currently Deployed (*n* = 36,460)			Previously Deployed (*n* = 57,521)		
	OR	(95% CI)	SRE^c^	OR	(95% CI)	SRE^c^	OR	(95% CI)	SRE^c^
Occupation									
Combat arms	1.1*	(1.0–1.2)	610	1.0	(0.9–1.2)	159	1.3*	(1.2–1.4)	358
Combat medic	1.5*	(1.3–1.6)	801	1.3*	(1.0–1.8)	208	1.2*	(1.0–1.5)	352
Other	1.0	–	546	1.0	–	155	1.0	–	283
χ^2^_2_	67.2*			4.5			32.8*		

^a^The sample of enlisted soldiers (*n* = 9650 cases, 153,523 control person-months) is a subset of the total sample (*n* = 193,617 person-months) from the Army STARRS Historical Administrative Data Study (HADS). Control person-months were assigned a weight of 200 to adjust for under-sampling

^b^Logistic regression models included gender, age at Army entry, current age, race/ethnicity, education, marital status, time in service (≤ 1 year, 2 years, 3–4 years, 5–10 years, >10 years), and military occupation. The models also included a dummy predictor variable for calendar month and year to control for secular trends

^c^SRE = standardized risk estimates (suicide attempters per 100,000 person-years) were calculated assuming other predictors were at their sample-wide means

*p < 0.05

We stratified the sample by time in service and examined occupation in similarly adjusted models. The association of occupation with suicide attempt was significant among soldiers with ≤10 years of service (≤ 1 year, χ^2^_2_ = 44.9, *p* < 0.0001; 2 years, χ^2^_2_ = 19.1, *p* < 0.0001; 3–4 years, χ^2^_2_ = 18.7, *p* < 0.0001; 5–10 years, χ^2^_2_ = 25.4, *p* < 0.0001), but not among those with more than 10 years of service (χ^2^_2_ = 3.7, *p* = 0.16). CA did not differ from AO during their first year of service, but had significantly higher odds of suicide attempt at 2 years (OR = 1.2 [95% CI: 1.0–1.3]), 3–4 years (OR = 1.2 [95% CI: 1.1–1.3]), and 5–10 years (OR = 1.4 [95% CI: 1.2–1.5]) of service. CA were at greatest risk in their first year of service (931/100,000 PY), with risk decreasing monotonically as time in service increased to more than 10 years (76/100,000 PY). In contrast, CM had significantly higher odds than AO throughout their first 4 years of service (≤ 1 year, OR = 1.5 [95% CI: 1.3–1.7]; 2 years, OR = 1.4 [95% CI: 1.2–1.7]; 3–4 years, OR = 1.3 [95% CI: 1.1–1.6]), but did not differ after 4 years of service. Risk was highest for CM in their first year of service (1313/100,000 PY) and decreased monotonically as time in service increased to more than 10 years (98/100,000 PY). AO had the lowest risk of suicide attempt, ranging from 868/100,000 PY during the first year of service to 69/100,000 PY in those with more than 10 years of service (Table 3; see Additional file 1: Table S8 for supplemental counts and rates by occupation and time in service). Importantly, in pairwise analyses CM had higher odds of suicide attempt than CA in the first year of service (OR = 1.4 [95% CI: 1.2–1.6]), which remained unchanged when limiting the analysis to males (OR = 1.4 [95% CI: 1.2–1.7]), but CM did not differ significantly from CA in subsequent years of service. When stratifying by occupation, time in service was associated with suicide attempt among CA (χ^2^_4_ = 88.8, *p* < 0.0001), CM (χ^2^_2_ = 45.4, *p* < 0.0001), and AO (χ^2^_2_ = 448.4, *p* < 0.0001). Among CA, odds of suicide attempt did not differ between the first and second year of service, but decreased significantly thereafter. Among CM and AO there was a consistent, significant decrease in the odds of suicide attempt as time in service increased (see Additional file 1: Table S9a and S9b for pairwise analyses by occupation and time in service).

**Table 3:** Multivariate associations of military occupation with suicide attempt among Regular Army enlisted soldiers stratified by time in service^a, b^

	Time in Service														
	≤ 1 Year (*n* = 25,786)			2 Years (*n* = 22,162)			3–4 Years (*n* = 36,155)			5–10 Years (*n* = 42,701)			>10 Years (*n* = 34,513)		
	OR	(95% CI)	SRE^c^	OR	(95% CI)	SRE^c^	OR	(95% CI)	SRE^c^	OR	(95% CI)	SRE^c^	OR	(95% CI)	SRE^c^
Occupation															
Combat arms	1.1	(1.0–1.2)	931	1.2*	(1.0–1.3)	657	1.2*	(1.1–1.3)	453	1.4*	(1.2–1.5)	279	1.1	(0.9–1.4)	76
Combat medic	1.5*	(1.3–1.7)	1313	1.4*	(1.2–1.7)	795	1.3*	(1.1–1.6)	494	1.2	(1.0–1.5)	253	1.4	(1.0–2.1)	98
Other	1.0	–	868	1.0	–	565	1.0	–	378	1.0	–	207	1.0	–	69
χ^2^_2_	44.9*			19.1*			18.7*			25.4*			3.7		

^a^The sample of enlisted soldiers (*n* = 9650 cases, 153,523 control person-months) is a subset of the total sample (*n* = 193,617 person-months) from the Army STARRS Historical Administrative Data Study (HADS). Control person-months were assigned a weight of 200 to adjust for under-sampling

^b^Logistic regression models included gender, age at Army entry, current age, race/ethnicity, education, marital status, deployment status (never, currently, or previously deployed), and military occupation. The models also included a dummy predictor variable for calendar month and year to control for secular trends

^c^SRE = standardized risk estimates (suicide attempters per 100,000 person-years) were calculated assuming other predictors were at their sample-wide means

*p < 0.05

### Monthly risk during the first year of service by occupation

**Fig. 1:**
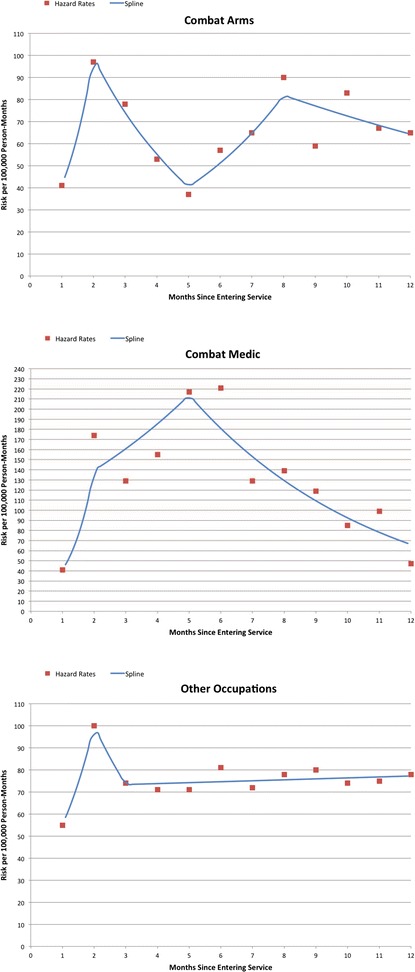
Monthly risk of suicide attempt by military occupation and among Regular Army enlisted soldiers in their first year of service^1,2^ ^1^The sample of enlisted soldiers in their first year of service (combat arms, *n*=6,853; combat medic, *n*=1,450; other, *n*=17,483) is a subset of the total sample of enlisted soldiers (*n*=163,173 person-months) from the Army STARRS Historical Administrative Data Study (HADS). Control person-months were assigned a weight of 200 to adjust for under-sampling. ^2^ Monthly risk based on hazard rates and linear spline models

Hazard functions and linear spline models suggest that the pattern of monthly suicide attempt risk during the first year of service varied by occupation (Fig. 1). CA had two distinct periods of elevated risk in the second month (97/100,000 person-months) and eighth month (90/100,000 person-months), with a period of decreased risk in between that dropped to a low of 37/100,000 person-months in the fifth month of service. Although risk among CM was also greatly elevated during the second month (174/100,000 person-months), it remained elevated through the fifth and sixth months (217–221/100,000 person-months) before beginning a steady decline to 47/100,000 person-months in the 12th month of service. Among AO there was a similar peak in risk during the second month (100/100,000 person-months), which then sharply declined and remained relatively stable from the third (74/100,000 person-months) to the 12th month (78/100,000 person-months).

## Discussion

We found that soldiers with a combat occupation had higher risk of suicide attempt than other soldiers, with the exception of SF. CA soldiers accounted for 26.0% of all enlisted suicide attempters, with an overall standardized risk of 417/100,000 PY (compared to 357/100,000 PY for other occupations). CM soldiers, while accounting for only 7.1% of attempters, had the highest standardized risk (504/100,000 PY). They also were more likely to attempt suicide than CA, a finding that persisted in males. The significant but modestly elevated odds for CA and CM versus AO persisted even after adjusting for previous mental health diagnosis. The unique aspects of military service, particularly during wartime, make it difficult to draw direct comparisons with the literature on suicide risk among civilian occupations. CA and CM soldiers share some similarities with police officers and emergency medical technicians, respectfully, two civilian occupational groups for which there is some evidence of elevated suicide risk [39].

Despite having an occupation that typically includes intense combat exposure over multiple deployments, SF accounted for only 16 suicide attempters from 2004 to 2009, with a standardized risk (102/100,000 PY) considerably lower than other occupations. The resilience of SF [21] may result from rigorous selection, intense training, [40] strong unit cohesion, [41] or psychological and biological characteristics [18–20]. Research on SF is lacking, and efforts to reduce suicide risk and mental healthcare stigma remain high priorities [42, 43]. Future Army STARRS studies with administrative data beyond 2009 will allow further examination of SF.

The higher risk for CA and CM varied by deployment status and time in service. CA and CM had higher risk if never or previously deployed, but occupation was unrelated to suicide attempt among those currently deployed. While there was a substantial difference in standardized risk in never deployed CM (801/100,000 PY) and CA (601/100,000 PY), there was virtually no difference in previously deployed (352 and 358/100,000 PY, respectively) [44].

The odds of suicide attempt were higher for CM than CA and AO during the first year of service. First-year CM had the highest standardized risk of all occupations across deployment status and time in service (1313/100,000 PY). Monthly hazard rates during the first year of service suggest that CA, CM, and AO have different patterns of risk during training. While all soldiers demonstrated rapidly increasing risk from the first to second month of service, the patterns deviated thereafter (approximately the end of basic training). First-year risk among CA was bimodal, whereas risk for CM remained elevated until the latter half of the first year. Training-related stressors may be particularly difficult to manage for those with pre-existing vulnerabilities, such as the considerable proportion of new soldiers who report a pre-enlistment history of suicidal behavior and mental disorders [45, 46].

After basic training CM undergo advanced training with high performance demands. A previous study found that mental health symptoms, including suicide ideation, increased over the course of CM training, and were associated with female gender and lower education [47] Although about 24% of CM in our sample were females and all CA soldiers were male, we found that the differential risk between first-year CM and CA persisted in males, suggesting that CM-CA differences are not due to gender composition. The proportion of soldiers with less than a high school education was substantially less for CM (9.8%) than CA (17.8%). Future research should examine the timing and relationships between lower education, poor training performance, and risk of suicide attempt among CM.

This study has five noteworthy limitations. First, the suicide attempt data are from administrative records. Although perhaps including the most serious cases, these records are subject to errors in clinician diagnosis or medical coding, and would not capture attempts that did not result in medical treatment. Second, our findings may not generalize to other periods of the Iraq and Afghanistan wars, or to other military conflicts. Third, we focused on a specific subset of occupations, but alternative categorizations are possible [48, 49]. In particular, there are a large number of occupations that fall under CA but are not as directly engaged in combat as a primary job function, e.g., combat engineers, which have elevated suicide deaths relative to the entire Army [13]. The organization of military occupations is not static, but changes over time based on the Army’s needs and strategic decisions [35, 36]. Fourth, we were not able to determine the degree to which the experiences and day-to-day responsibilities of individual soldiers corresponded to their assigned occupational code. Most significantly, these administrative data did not allow assessment of combat exposure. While all occupations considered have a high likelihood of direct combat exposure, actual exposure will vary. Finally, differences across deployment status and time in service are not evidence of within-person changes in suicide attempt risk over time, as the composition of these groups is affected by the non-random nature of deployment and Army retention and attrition [50].

## Conclusions

Our findings suggest that occupation may assist in identifying the sub-populations and timing of elevated risk for suicide attempt in the U.S. Army, but the individual risk associated with occupation remains modest. Although we found that combat roles are generally associated with elevated risk, the importance of occupation begins during enlistment. Soldiers often choose their career field, but the Army also assigns occupations based on a soldier’s aptitude or performance; therefore soldiers with the same occupation may share similar characteristics (e.g., backgrounds, traits, abilities) that could influence adjustment and mental health [51–55]. To distinguish the extent to which occupational stressors uniquely contribute to suicide risk, it is important for future studies to parse out the effects of individual characteristics that predict career selection, exposures, resilience, and suicide risk. It is also important for future studies to consider how access to lethal means, mental health stigma, and help-seeking behavior vary across occupations. This may be possible in future Army STARRS analyses using longitudinal follow-up surveys and new administrative data systems with potentially important predictors [56].

## Additional file

Additional file 1: Contains tables with additional methodological information and statistical results. (PDF 1868 kb)

## Abbreviations

AFMETS: Armed Forces Medical Examiner Tracking System
AO: all other (referring to all other occupations)
CA: combat arms
CI: confidence interval
CM: combat medic
CTS: Contingency Tracking System
DCIPS: Defense Casualty Information Processing System
DEERS: Defense Enrollment Eligibility Reporting System
DMDC: Defense Manpower Data Center
DOD: Department of Defense
DODSER: Department of Defense Suicide Event Report
HADS: Historical Administrative Data Study
ICD-9-CM: International Classification of Diseases, Ninth Revision–Clinical Modification
MDR: Military Health System Data Repository
MOS: military occupational specialty
NIH: National Institutes of Health
NIMH: National Institute of Mental Health
OR: odds ratio
Pop %: percent of the population
PY: person-years
SAS: Statistical Analysis System
SF: special forces
SRE: standardized risk estimate
STARRS: Study to Assess Risk and Resilience in Servicemembers
TMDS: Theater Medical Data Store
TRAC2ES: Transportation Command Regulating And Command And Control Evacuation System
US: United States
USAPHC: United States Army Public Health Command
VA: Veterans Affairs
