# Supplementary material for: Suicide attempts in U.S. Army combat arms, special forces and combat medics
Source: BMC Psychiatry. 2017 May 25;17:194. doi: 10.1186/s12888-017-1350-y (PMC5445296; doi:10.1186/s12888-017-1350-y)
Supplement: Additional file 1: — Contains tables with additional methodological information and statistical results. (PDF 1868 kb) [file 12888_2017_1350_MOESM1_ESM.pdf]

Additional File 1:
Supplemental Tables and Information

Table S1. List and Brief Descriptions of Administrative Data Systems Included in the 2004-2009 Army STARRS Historical Administrative Data Study (HADS).

Database Acronym	Description
AFMETS	ARMED FORCES MEDICAL EXAMINER TRACKING SYSTEM (AFMETS): Variables include manner of death and cause of death, including self-inflicted.
DCIPS	DEFENSE CASUALTY INFORMATION PROCESSING SYSTEM (DCIPS): Casualty data system for Army, Navy, Air Force, and Marines. It contains information on all casualties including deceased active duty members (and cause of death).
DMDC/CTS	DEFENSE MANPOWER DATA CENTER (DMDC) / CONTINGENCY TRACKING SYSTEM (CTS): Collection of activation, mobilization, and deployment data. Provides information to DoD decision makers and includes a CTS Deployment File used for tracking the location of deployed personnel.
DMDC/Master Personnel & DMDC/Transaction files	DEFENSE MANPOWER DATA CENTER (DMDC) / MASTER PERSONNEL & TRANSACTION FILES: The Active Duty Master File provides an inventory of all individuals on active duty (excluding reservists on active duty for training) at a point in time. It is a standardized and centralized database of present and past members of the active duty force. Personal data elements include social security number, education level, home of record, date of birth, marital status, number of dependents, race, ethnic group, and name. Military data elements include Service, pay grade, Armed Forces Qualification Test percentile (enlisted only), source of commission (officers only), military primary duty and secondary occupation, Unit Identification Code, months of service, duty location, Estimated Termination of Service date, basic active service date, date of current rank, pay entry base date, foreign language ability, and major command code.
DODSER	DEPARTMENT OF DEFENSE SUICIDE EVENT REPORT (DODSER): Provides risk and protective factor information for suicide events. This file contains non-fatal attempts, completed suicides, and suicide ideation cases.
MDR	MILITARY HEALTH SYSTEM DATA REPOSITORY (MDR): This database contains information about medical, dental, pharmaceutical, and ancillary claims data for both in network and purchased care as well as both inpatient and outpatient treatment. Data are collected on both Army personnel and their beneficiaries.
DMDC/DEERS	DEFENSE MANPOWER DATA CENTER (DMDC) / DEFENSE ENROLLMENT ELIGIBILITY REPORTING SYSTEM (DEERS): A DoD PDR containing personnel data and data related to DoD affiliation, benefits, employment, and pay.
TMDS	THEATER MEDICAL DATA STORE (TMDS): Used to track, analyze, view and manage Soldier medical treatment information recorded in the theater of operations. Features of TMDS: accessibility and visibility of service members' deployed medical records, outpatient and inpatient treatment records created in theater facilities, treatment records from other applications, reports on movement of patients, patient status and injury/illnesses.
TRAC2ES	TRANSCOM REGULATING AND COMMAND AND CONTROL EVACUATION SYSTEM (TRAC2ES): A tracking system for all medical transfers across the world for all DOD services.

Table S2. List of Military Occupational Specialties (MOS) Included in the 2004-2009 Army STARRS Historical Administrative Data Study (HADS).

**Combat Arms**		
	E11B	Infantryman
	E11C	Indirect Fire Infantryman
	E11Z	Infantry Senior Sergeant
	E13B	Cannon Crewmember
	E13E	Cannon Fire Direction Specialist
	E13F	Fire Support Specialist
	E13S	Field Artillery Surveyor
	E19D	Cavalry Scout
	E19K	M1 Armor Crewman
	E19Z	Armor Senior Sergeant
**Special Forces**		
	E00D	Special Duty Assignment
	E18B	Special Forces Weapons Sergeant
	E18C	Special Forces Engineer Sergeant
	E18E	Special Forces Communications Sergeant
	E18F	Special Forces Assistant Operations and Intelligence Sergeant
	E18Z	Special Forces Senior Sergeant
**Combat Medic**		
	E68W	Health Care Specialist
	E91W	Health Care Specialist
**All Other (i.e., not included in the Combat Arms, Special Forces, or Combat Medic categories above)**		
Air Traffic Controller		
	E15P	Aviation Operations Specialist
	E15Q	Air Traffic Control (ATC) Operator
Chaplains Assistant/Chaplain		
	E56M	Chaplain Assistant
Combat Engineer		
	E12B	Combat Engineer
	E12Z	Combat Engineering Senior Sergeant
	E21B	Combat Engineer
	E21Z	Combat Engineering Senior Sergeant
Communication		
	E25B	Information Technology Specialist
	E25C	Radio Operator-Maintainer
	E25D	Telecommunications Operator-Maintainer
	E25E	Electromagnetic Spectrum Manager
	E25F	Network Switching Systems Operator-Maintainer
	E25L	Cable Systems Installer-Maintainer
	E25M	Multimedia Illustrator

E25N	Nodal Network Systems Operator-Maintainer
E25P	Microwave Systems Operator-Maintainer
E25Q	Multichannel Transmission Systems Operator-Maintainer
E25R	Visual Information Equipment Operator-Maintainer
E25S	Satellite Communications Systems Operator-Maintainer
E25T	Satellite/Microwave Systems Chief
E25U	Signal Support Systems Specialist
E25V	Combat Documentation/Production Specialist
E25W	Telecommunications Operations Chief
E25Y	Information Systems Chief
E25Z	Visual Information Operations Chief
E31F	Network Switching Systems Operator-Maintainer
E31L	Cable Systems Installer-Maintainer
E31P	Microwave Systems Operator-Maintainer
E31R	Multichannel Transmission Systems Operator-Maintainer
E31S	Satellite Communications Systems Operator-Maintainer
E31T	Satellite/Microwave Systems Chief
E31U	Signal Support Systems Specialist
E31W	Telecommunications Operations Chief
E31Z	Senior Signal Sergeant
E74B	Information Systems Operator-Analyst
E74C	Telecommunications Operator-Maintainer
E74Z	Information Systems Chief

Construction

E21C	Bridge Crewmember
E21E	Construction Equipment Operator
E21F	Crane Operator
E21H	Construction Engineering Supervisor
E21J	General Construction Equipment Operator
E21K	Plumber
E21M	Firefighter
E21N	Construction Equipment Supervisor
E21P	Prime Power Production Specialist
E21R	Interior Electrician
E21S	Topographic Surveyor
E21T	Technical Engineer
E21U	Topographic Analyst
E21V	Concrete and Asphalt Equipment Operator
E21W	Carpentry and Masonry Specialist
E21Y	Geospatial Engineer
E62B	Construction Equipment Repairer
E62H	Concrete & Asphalt Equipment Operator

Food Personnel

E92G	Food Service Specialist

Health Care Personnel (Excluding Combat Medic)

E18D	Special Forces Medical Sergeant
E68A	Biomedical Equipment Specialist

E68D	Operating Room Specialist
E68E	Dental Specialist
E68H	Optical Laboratory Specialist
E68J	Medical Logistics Specialist
E68K	Medical Laboratory Specialist
E68M	Nutrition Care Specialist
E68P	Radiology Specialist
E68Q	Pharmacy Specialist
E68R	Veterinary Food Inspection Specialist
E68S	Preventive Medicine Specialist
E68T	Animal Care Specialist
E68V	Respiratory Specialist
E68Z	Chief Medical NCO
E91A	Medical Equipment Repairer
E91D	Operating Room Specialist
E91E	Dental Specialist
E91G	Patient Administration Specialist
E91H	Optical Laboratory Specialist
E91J	Medical Logistics Specialist
E91K	Medical Laboratory Specialist
E91M	Nutrition Care Specialist
E91P	Radiology Specialist
E91Q	Pharmacy Specialist
E91R	Veterinary Food Inspection Specialist
E91S	Preventive Medicine Specialist
E91T	Animal Care Specialist
E91V	Respiratory Specialist
E91Z	Chief Medical NCO

Intelligence/Interrogators

E15W	Unmanned Aerial Vehicle (UAV) Operator
E33W	Military Intelligence Systems Maintainer/Integrator
E35F	Intelligence Analyst
E35G	Imagery Analyst
E35K	Unmanned Aerial Vehicle (UAV) Operator
E35L	Counter Intelligence Agent
E35M	Human Intelligence Collector
E35N	Signals Intelligence Analyst
E35P	Cryptologic Linguist
E35S	Signals Collector/Analyst
E35W	EW/SIGINT Specialty (Linguist) Training Accession Reporting Code
E35Y	Chief Counterintelligence/Human Intelligence Sergeant
E35Z	Signals Intelligence (SIGINT) Senior Sergeant/SIGINT Chief
E96B	Intelligence Analyst
E96D	Imagery Analyst
E96H	Common Ground Station Operator
E96R	Ground Surveillance Systems Operator
E96U	Unmanned Aerial Vehicle Operator
E96Z	Intelligence Senior Sergeant
E97B	Counter-Intelligence Analyst

	E97E	Human Intelligence Collector
	E97Z	Counterintelligence/Human Intelligence, Senior Sergeant
	E98C	Signals Intelligence Analyst
	E98G	Cryptologic Communications Interceptor/Locator
	E98H	Communications Interceptor/Locator
	E98J	Electronic Intelligence Interceptor/Analyst
	E98K	Signals Collection/Identification Analyst
	E98P	Multi-Sensor Operator
	E98Y	Signals Collector/Analyst
	E98Z	Signals Intelligence (Electronic Warfare) Senior Sergeant/Chief
Law Enforcement/Military Police		
	E27D	Paralegal Specialist
	E31B	Military Police
	E31D	CID Special Agent
	E31E	Internment/Resettlement Specialist
	E95B	Military Police
Missiles/Rockets Operators (Combat)		
	E13C	Tactical Automated Fire Control Systems Specialist
	E13D	Field Artillery Automated Tactical Data System Specialist
	E13M	Multiple Launch Rocket System (MLRS) Crewmember
	E13R	Field Artillery (FA) Firefinder Radar Operator
	E13W	Field Artillery Meteorological Crewmember
	E14E	PATRIOT Fire Control Enhanced Operator/Maintainer
	E14J	Air Defense Command, Control, Communications, Computers and Intelligence Tactical Operations Center Enhanced Operator/Maintainer
	E14R	BRADLEY LINEBACKER Crewmember
	E14S	Air and Missile Defense (AMD) Crewmember
	E14T	PATRIOT Launching Station Enhanced Operator/Maintainer
	E14Z	Air Defense Artillery Senior Sergeant
Missiles/Rockets Operators (Non-Combat)		
	E13P	Multiple Launch Rocket System (MLRS) Operations/Fire Direction Specialist
	E13Z	Field Artillery Senior Sergeant
Mortuary Affairs Personnel		
	E92M	Mortuary Affairs Specialist
Motor Transport		
	E88H	Cargo Specialist
	E88K	Watercraft Operator
	E88L	Watercraft Engineer
	E88M	Motor Transport Operator
	E88N	Transportation Management Coordinator
	E88Z	Transportation Senior Sergeant
Nuclear, Chemical, Biological Operations Specialist		
	E54B	Chemical Operations Specialist
	E55B	Ammunition Specialist

	E55D	Explosive Ordnance Disposal Specialist
	E74D	Chemical, Biological, Radiological, and Nuclear (CBRN) Specialist
	E89A	Ammunition Stock Control and Accounting Specialist
	E89B	Ammunition Specialist
	E89D	Explosive Ordnance Disposal Specialist
PsyOps		
	E37F	Psychological Operations Specialist
Recruiter		
	E79R	Recruiter
	E79S	Career Counselor
	E79T	Recruiting and Retention NCO (Army National Guard of the United States)
	E79V	Army Reserve Career Counselor (Army Reserve)
Repair/Maintenance		
	E15B	Aircraft Powerplant Repairer
	E15D	Aircraft Powertrain Repairer
	E15F	Aircraft Electrician
	E15G	Aircraft Structural Repairer
	E15H	Aircraft Pneudraulics Repairer
	E15J	OH-58D/ARH Armament/Electrical/Avionics Systems Repairer
	E15K	Aircraft Components Repair Supervisor
	E15M	UH-1 Helicopter Repairer (RC)
	E15N	Avionic Mechanic
	E15R	AH-64 Attack Helicopter Repairer
	E15S	OH-58D/ARH Helicopter Repairer
	E15T	UH-60 Helicopter Repairer
	E15U	CH-47 Helicopter Repairer
	E15V	Observation/Scout Helicopter Repairer (RC)
	E15Y	AH-64D Armament/Electrical/Avionics Systems Repairer
	E15Z	Aircraft Maintenance Senior Sergeant
	E27E	Land Combat Electronic Missile System Repairer
	E27M	Multiple Launch Rocket System (MLRS) Repairer
	E27T	AVENGER System Repairer
	E27Z	Missile Systems Maintenance Chief
	E31C	Radio Operator-Maintainer
	E35A	Land Combat Electronics Missile System Repairer
	E35D	Air Traffic Control Equipment Repairer
	E35E	Radio and Communications Security (COMSEC) Repairer
	E35F	Special Electronic Devices Repairer
	E35H	TMDE Maintenance Support Specialist
	E35J	Computer/Automation Systems Repairer
	E35K	APACHE Attack Helicopter Systems Repairer
	E35L	Avionic Communications Equipment Repairer
	E35M	Radar Repairer
	E35N	Wire Systems Equipment Repairer
	E35P	Multiple Launch Rocket System (MLRS) Repairer
	E35R	Avionic Systems Repairer
	E35S	PATRIOT System Repairer

E35T	AVENGER System Repairer
E35V	Electronic and Missile Systems Maintenance Chief
E35W	Electronic Maintenance Chief
E35Y	Integrated Family of Test Equipment Operator/Maintainer
E35Z	Senior Electronics Maintenance Chief
E39B	Automatic Test Equipment Operator/Maintainer
E44B	Metal Worker
E44E	Machinist
E45B	Small Arms/Artillery Repairer
E45D	Self-Propelled Field Artillery Turret Mechanic
E45E	M1 ABRAMS Tank Turret Mechanic
E45G	Fire Control Repairer
E45K	Armament Repairer
E51R	Interior Electrician
E51Z	General Engineering Supervisor
E52C	Utilities Equipment Repairer
E52D	Power-Generation Equipment Repairer
E63A	M1 ABRAMS Tank System Maintainer
E63B	Wheeled Vehicle Mechanic
E63D	Artillery Mechanic
E63E	M1 ABRAMS Tank System Mechanic
E63G	Fuel & Electrical System Repairer
E63H	Track Vehicle Repairer
E63J	Quartermaster and Chemical Equipment Repairer
E63M	BRADLEY Fighting Vehicle System Maintainer
E63S	Heavy Wheel Vehicle Mechanic
E63T	BRADLEY Fighting Vehicle System Mechanic
E63W	Wheel Vehicle Repairer
E63Y	Track Vehicle Mechanic
E63Z	Mechanical Maintenance Supervisor
E67N	UH-1 Helicopter Repairer
E67R	AH-64 Attack Helicopter Repairer
E68B	Aircraft Powerplant Repairer
E68G	Aircraft Structural Repairer
E68J	Aircraft Armament/Missile Systems Repairer (RC)
E68N	Avionic Mechanic
E91A	M1 Abrams Tank System Maintainer
E91B	Wheeled Vehicle Repairer
E91C	Utilities Equipment Repairer
E91D	Power Generation Equipment Repairer
E91E	Allied Trades Specialist
E91F	Small Arms/Artillery Repairer
E91G	Fire Control Repairer
E91H	Tracked Vehicle Repairer
E91J	Quartermaster and Chemical Equipment Repairer
E91K	Armament Repairer
E91L	Construction Equipment Repairer
E91M	BRADLEY Fighting Vehicle System Maintainer
E91P	Artillery Mechanic
E91W	Metal Worker

E94A	Land Combat Electronic Missile System Repairer
E94D	Air Traffic Control Equipment Repairer
E94E	Radio and Communications Security (COMSEC) Repairer
E94F	Computer/Detection Systems Repairer
E94H	Test, Measurement, and Diagnostic Equipment (TMDE) Maintenance Support Specialist
E94K	APACHE Attack Helicopter Systems Repairer
E94L	Avionic Communications Equipment Repairer
E94M	Radar Repairer
E94P	Multiple Launch Rocket System (MLRS) Repairer
E94R	Avionic and Survivability Equipment Repairer
E94S	PATRIOT System Repairer
E94T	AVENGER System Repairer
E94V	Electronic and Missile Systems Maintenance Chief
E94W	Electronic Maintenance Chief
E94Y	Integrated Family of Test Equipment (IFTE) Operator and Maintainer
E94Z	Senior Electronic Maintenance Chief

Trainee

E09B	Trainee
E09C	Trainee, Language
E09R	Simultaneous Management Program Participant
E09S	Commissioned Officer Candidate
E09W	Warrant Officer Candidate
E11X	Infantryman
E15X	AH-64A Armament/Electrical Systems Repairer
E18X	Special Forces Weapons Sergeant
E21X	General Engineering Supervisor
E25X	Senior Signal Sergeant
E27X	PATRIOT System Repairer
E35X	Intelligence Senior Sergeant/Chief Intelligence Sergeant
E52X	Special Purpose Equipment Repairer
E63X	Maintenance Supervisor
E68X	AH-64A Armament/Electrical Systems Repairer
E91X	Maintenance Supervisor
E91X	Mental Health Specialist
E94X	Senior Missile Systems Maintainer
E98X	EW/SIGINT Specialty (Linguist) (Reporting Code)

Undefined MOS (MOS not in 2004-09 database or MOS with invalid date range)

E00R
E09D
E11H
E29J
E33T
E35L
E35M
E35S
E35Z
E43E

E43M
E68J
E68K
E68S
E71D
E71G
E75E
E76J
E76P
E91D
E91E
E91J
E91P
E94B

MOS not in any of the above categories)

E00Z	Command Sergeant Major
E02A	Army Bandperson
E02B	Cornet or Trumpet Player
E02C	Euphonium Player
E02D	French Horn Player
E02E	Trombone Player
E02F	Tuba Player
E02G	Flute or Piccolo Player
E02J	Clarinet Player
E02L	Saxophone Player
E02M	Percussion Player
E02N	Keyboard Player
E02S	Special Bands Member
E02T	Guitar Player
E02U	Electric Bass Player
E02Z	Bands Senior Sergeant
E09L	Interpreter/Translator
E21D	Diver
E21L	Lithographer
E35H	Common Ground Station (CGS) Analyst
E36B	Financial Management Technician
E38A	Civil Affairs Specialist (RC)
E38B	Civil Affairs Specialist
E42A	Human Resources Specialist
E42F	Human Resources Information Systems Management Specialist
E42L	Administrative Specialist
E42R	Army Bandperson
E42S	Special Band Member
E44C	Financial Management Technician
E46Q	Public Affairs Specialist
E46R	Public Affairs Broadcast Specialist
E46Z	Chief Public Affairs NCO
E51C	Acquisition, Logistics & Technology (AL&T) Contracting NCO
E57E	Landry & Bath Specialist

E71L	Administrative Specialist
E73C	Finance Specialist
E73D	Accounting Specialist
E73Z	Finance Senior Sergeant
E75H	Personnel Services Specialist
E77F	Petroleum Supply Specialist
E92A	Automated Logistical Specialist
E92F	Petroleum Supply Specialist
E92L	Petroleum Laboratory Specialist
E92R	Parachute Rigger
E92S	Shower/Laundry and Clothing Repair Specialist
E92W	Water Treatment Specialist
E92Y	Unit Supply Specialist
E92Z	Senior Noncommissioned Logistician

Table S3: International Classification of Diseases, Ninth Revision–Clinical Modification (ICD-9-CM) Codes Used to Identify Mental Disorders.

Included Mental Health Diagnoses	ICD-9-CM Codes
Adjustment Disorder	309, .29, .3, .4, .82, .83, .89, .9
Dysthymic Disorder/ Neurasthenia/ Depression NOS	296.82, .90, .99
	300.4, .5
	309.0, .1
	311, .0, .1
	313.1
Major Depression	296.2, .20, .21, .22, .23, .24, .25, .26, .3, .30, .31, .32, .33, .34, .35, .36
Bipolar Disorder	296.00, .01, .02, .03, .04, .05, .06, .10, .11, .12, .13, .14, .15, .16, .40, .41, .42, .43, .44, .45, .46, .50, .51, .52, .53, .54, .55, .56, .60, .61, .62, .63, .64, .65, .66, .7, .80, .81, .89
	301.13
Anxiety State/ Anxiety Disorder	300, .00, .01, .02, .09, .20, .21, .22, .23, .29, .3
	309.21, .24, .28
	313.0, .21, .22, .23
Post-Traumatic Stress Disorder	309.81
Attention-Deficit/Hyperactivity Disorder/ Learning Disorders	314.0, .00, .01, .1, .2, .8, .9
	315.00, .01, .02, .09, .1, .2, .3, .31, .32, .34, .39, .4, .5, .8, .9
Conduct Disorder/ Oppositional Defiant Disorder	301.7
	312.4, .8, .81, .82, .89, .9
	313.81
	V62.83
Eating Disorders	307.1, .50, .51, .59
Other Impulse Control Disorders	312.00, .01, .02, .03, .10, .11, .12, .13, .20, .21, .22, .23, .3, .30, .31, .32, .33, .34, .35, .39
Alcohol Induced Mental Disorders/ Alcohol Dependence/ Alcohol Abuse	291.0, .1, .2, .3, .4, .5, .8, .81, .82, .89, .9
	303.00, .01, .02, .03, .9, .90, .91, .92, .93
	305, .0, .00, .01, .02, .03

Drug Induced Mental Disorders	292
Non-Dependent Drug Abuse	305.2, .20, .21, .22, .23, .3, .30, .31, .32, .33, .4, .40, .41, .42, .43, .5, .50, .51, .52, .53, .6, .60, .61, .62, .63, .7, .70, .71, .72, .73, .8, .80, .81, .82, .83, .9, .90, .91, .92, .93
Drug dependence	304
Personality Disorders	301.0, .1, .10, .11, .12, .20, .21, .22, .3, .4, .50, .51, .59, .6, .8, .80, .81, .82, .83, .84, .89, .9
Non-Affective Psychosis	295.00, .01, .02, .03, .04, .05, .10, .11, .12, .13, .14, .15, .20, .21, .22, .23, .24, .25, .30, .31, .32, .33, .34, .35, .40, .41, .42, .43, .44, .45, .50, .51, .52, .53, .54, .60, .61, .62, .63, .64, .65, .70, .71, .72, .73, .74, .75, .80, .81, .82, .83, .84, .85, .90, .91, .92, .93, .94, .95
	297.0, .1, .2, .3, .8, .9
	298.0, .1, .2, .3, .4, .8, .9, .90
Somatoform/ Dissociative Disorders	300.10, .11, .12, .13, .14, .15, .16, .19, .6, .7, .80, .81, .82, .89
	306.0, .1, .2, .3, .4, .50, .51, .52, .53, .59, .6, .7, .8, .9
	307.54, .80, .81, .89
Organic Mental Disorders	290.0, .10, .11, .12, .13, .20, .21, .3, .40, .41, .42, .43, .8, .9
	293.0, .1, .81, .82, .83, .84, .89, .89, .9
	294.0, .1, .10, .11, .8, .9
	307.20, .21, .22, .23, .3
	310.0, .8, .9
	317
	318.0, .1, 2
	319
Sexual Disorders	302, .0, .1, .2, .3, .4, .50, .51, .52, .53, .6, .70, .71, .72, .73, .74, .75, .76, .79, .81, .82, .83, .84, .85, .89, .9
Sleep Disorders	307.4, .40, .41, .42, .43, .44, .45, .46, .47, .48, .49
Other Mental Disorders/ Mental Illness	292.85
	299.00, .01, .10, .80, .81, .90, .91
	300.9
	307.0, .52, .53, .6, .7, .9
	309.22
	310.1

	313.3, .82, .89, .9
	316
Traumatic Stress	308, .0, .1, .2, .3, .4, .9

**Excluded Mental Health Diagnoses**	**ICD-9-CM Codes**
Postconcussion Syndrome	310.2
Tobacco Use Disorder	305.1, .10, .11, .12, .13
Symptoms, Signs, and Ill-Defined Conditions, Mental	797
	798, .0, .1, .2, .9
	799, .0, .01, .02, .1, .2, .21, .22, .23, .24, .25, .29, .3, .4, .8, .81, .82, .89, .9
Stressors/ Adversities	V40.0, .00, .1, .2, .20, .3, .30, .9, .90
	V61, .0, .01, .02, .03, .04, .05, .06, .07, .08, .09, .2, .20, .21, .22, .23, .24, .29, .3, .4, .41, .42, .49, .8, .9
	V62, .0, .1, .1 0, .2, .20, .21, .22, .29, .3, .4, .5, .8, 80, .81, .810, .811, .812, .82, .89, .9, .90
	V69.4, .5, .9
Marital Problems	V61.1, .10, .11, .12
Prior History of Mental Disorders	V11.0, .1, .2, .3, .8, .80, .9, .90
	V66.3
	V67.3
Indicator of Impulsivity and Risky Behavior	V69.2, .3
Self-Damaging Behavior	V69.8

Table S4. Multivariate association of military occupation with suicide attempt among Regular Army enlisted soldiers, adjusting for socio-demographics, service-related variables, and prior mental health diagnosis.[1, 2]

	OR	(95% CI)	Cases (n)	Total (n)[3]	Rate[4]	Pop %[5]	SRE[6]
**Occupation**							
Combat arms	1.2*	(1.1–1.3)	2,506	7,159,106	420	23.3	427
Special forces	0.5*	(0.3–0.7)	16	368,016	52	1.2	162
Combat medic	1.3*	(1.2–1.4)	682	1,470,882	556	4.8	464
Other	1.0	–	6,446	21,716,246	356	70.7	355
χ^2_3		96.2*					

[1]The sample of enlisted soldiers (n=9,650 cases, 153,523 control person-months) is a subset of the total sample (n=193,617 person-months) from the Army STARRS Historical Administrative Data Study (HADS). Control person-months were assigned a weight of 200 to adjust for under-sampling.

[2]Logistic regression models included gender, age at Army entry, current age, race/ethnicity, education, marital status, time in service (≤ 1 year, 2 years, 3-4 years, 5-10 years, >10 years), deployment status (never, currently, or previously deployed), prior mental health diagnosis, and military occupation. The models also included a dummy predictor variable for calendar month and year to control for secular trends.

[3]Total includes both cases (i.e., soldiers with a suicide attempt) and control person-months.

[4]Rate per 100,000 person-years, calculated based on n_1/n_2, where n_1 is the unique number of soldiers within each category and n_2 is the annual number of person-*years*, not person-*months*, in the population (n=3.08 million).

[5]Pop % = percent of the population of enlisted soldier.

[6]SRE = Standardized risk estimates (suicide attempters per 100,000 person-years) were calculated assuming other predictors were at their sample-wide means.

*$p < 0.05$

Table S5. Multivariate association of military occupation with suicide attempt among male and female Regular Army enlisted soldiers, adjusting for socio-demographics, service-related variables, and prior mental health diagnosis.[1, 2]

	Males			Females		
	OR	(95% CI)	SRE[3]	OR	(95% CI)	SRE[3]
**Occupation**						
Combat arms	1.2*	(1.2–1.3)	370	–	–	–
Special forces	0.5*	(0.3–0.7)	139	–	–	–
Combat medic	1.3*	(1.2–1.4)	389	1.3*	(1.2–1.5)	884
Other	1.0	–	303	1.0	–	675
		$\chi^2_3 = 83.2$*			$\chi^2_1 = 18.6$*	

[1]The sample of enlisted soldiers (n=9,650 cases, 153,523 control person-months) is a subset of the total sample (n=193,617 person-months) from the Army STARRS Historical Administrative Data Study (HADS). Control person-months were assigned a weight of 200 to adjust for under-sampling.

[2]Logistic regression models included gender, age at Army entry, current age, race/ethnicity, education, marital status, time in service (≤ 1 year, 2 years, 3-4 years, 5-10 years, >10 years), deployment status (never, currently, or previously deployed), prior mental health diagnosis, and military occupation. The models also included a dummy predictor variable for calendar month and year to control for secular trends.

[3]SRE = standardized risk estimates (suicide attempters per 100,000 person-years) were calculated assuming other predictors were at their sample-wide means.

*$p < 0.05$

Table S6. Counts and rates of suicide attempt by military occupation among Regular Army enlisted soldiers stratified by deployment status.[1]

	Deployment Status														
	Never Deployed (n = 67,336)					Currently Deployed (n = 36,460)					Previously Deployed (n = 57,521)				
	Cases (n)	Total (n)[2]	Rate[3]	Pop %[4]	SRE[5]	Cases (n)	Total (n)[2]	Rate[3]	Pop %[4]	SRE[5]	Cases (n)	Total (n)[2]	Rate[3]	Pop %[4]	SRE[5]
**Occupation**															
Combat arms	1,286	2,374,686	650	19.3	610	262	2,053,262	153	28.9	159	958	2,731,158	421	25.0	358
Combat medic	480	667,680	863	5.4	801	59	304,259	233	4.3	208	143	498,943	344	4.6	352
Other	4,117	9,254,117	534	75.3	546	619	4,747,419	157	66.8	155	1,710	7,714,710	266	70.5	283

[1]The sample of enlisted soldiers (n=9,650 cases, 153,523 control person-months) is a subset of the total sample (n=193,617 person-months) from the Army STARRS Historical Administrative Data Study (HADS). Control person-months were assigned a weight of 200 to adjust for under-sampling.

[2]Total includes both cases (i.e., soldiers with a suicide attempt) and control person-months.

[3]Rate per 100,000 person-years, calculated based on n_1/n_2, where n_1 is the unique number of soldiers within each category and n_2 is the annual number of person-*years*, not person-*months*, in the population (n=3.08 million).

[4]Pop % = Population percent.

[5]SRE = Standardized risk estimates (suicide attempters per 100,000 person-years) were calculated assuming other predictors were at their sample-wide means. SREs were calculated based on logistic regression models that included gender, age at entry into the Army, current age, race/ethnicity, education, marital status, time in service (≤ 1 year, 2 years, 3-4 years, 5-10 years, >10 years), deployment status (never, currently, or previously deployed), and military occupation. The models also included a dummy predictor variable for calendar month and year to control for secular trends.

Table S7a. Pairwise tests examining multivariate associations of deployment status with suicide attempt among Regular Army enlisted soldiers stratified by military occupation.[1,2]

Deployment status	Occupation					
	Combat arms		Combat medic		Other	
	OR	(95% CI)	OR	(95% CI)	OR	(95% CI)
Never deployed vs. currently deployed	3.3*	(2.8–3.8)	1.9*	(1.4–2.6)	2.1*	(1.9–2.3)
Previously deployed vs. currently deployed	3.8*	(3.3–4.4)	2.4*	(1.7–3.2)	2.5*	(2.3–2.8)
Previously deployed vs. never deployed	1.2*	(1.0–1.3)	1.2	(1.0–1.6)	1.2*	(1.1–1.3)
χ^2_2	367.0*		29.8*		378.1*	

eTable 7b. Pairwise tests examining multivariate associations of military occupation with suicide attempt among Regular Army enlisted soldiers stratified by deployment status.[1,2]

Occupation	Deployment status					
	Never deployed		Currently deployed		Previously deployed	
	OR	(95% CI)	OR	(95% CI)	OR	(95% CI)
Combat arms vs. other	1.1*	(1.0–1.2)	1.0	(0.9–1.2)	1.3*	(1.2–1.4)
Combat medic vs. other	1.5*	(1.3–1.6)	1.3*	(1.0–1.8)	1.2*	(1.0–1.5)
Combat medic vs. combat arms	1.3*	(1.2–1.5)	1.3	(1.0–1.8)	1.0	(0.8–1.2)
χ^2_2	67.2*		4.5		32.8*	

[1]The sample of enlisted soldiers (n=9,650 cases, 153,523 control person-months) is a subset of the total sample (n=193,617 person-months) from the Army STARRS Historical Administrative Data Study (HADS). Control person-months were assigned a weight of 200 to adjust for under-sampling.

[2]Logistic regression models included gender, age at Army entry, current age, race/ethnicity, education, marital status, time in service (≤ 1 year, 2 years, 3-4 years, 5-10 years, >10 years), deployment status (never, currently, or previously deployed), and military occupation. The models also included a dummy predictor variable for calendar month and year to control for secular trends.

*$p < 0.05$

Table S8. Counts and rates of suicide attempts by military occupation among Regular Army enlisted soldiers stratified by time in service.[1]

	Times in service									
	≤ 1 year (n = 25,786)					2 years (n = 22,162)				
	Cases (n)	Total (n)[2]	Rate[3]	Pop %[4]	SRE[5]	Cases (n)	Total (n)[2]	Rate[3]	Pop %[4]	SRE[5]
**Occupation**										
Combat arms	800	1,211,400	793	27	931	545	1,136,945	575	28.2	657
Combat medic	301	230,101	1,570	5.1	1,313	133	186,533	856	4.6	795
Other	2,297	3,039,497	907	67.8	868	1,334	2,708,534	591	67.2	565

	3–4 years (n = 36,155)					5-10 years (n = 42,701)				
	Cases (n)	Total (n)[2]	Rate[3]	Pop %[4]	SRE[5]	Cases (n)	Total (n)[2]	Rate[3]	Pop %[4]	SRE[5]
**Occupation**										
Combat arms	673	1,847,073	437	27.3	453	409	1,720,809	285	20.9	279
Combat medic	131	323,531	486	4.8	494	87	394,287	265	4.8	253
Other	1,473	4,607,273	384	68	378	1,043	6,118,843	205	74.3	207

	>10 years (n = 34,513)				
	Cases (n)	Total (n)[2]	Rate[3]	Pop %[4]	SRE[5]
**Occupation**					
Combat arms	79	1,242,879	76	18.2	76
Combat medic	30	336,430	107	4.9	98
Other	299	5,242,099	68	76.8	69

[1]The sample of enlisted soldiers (n=9,650 cases, 153,523 control person-months) is a subset of the total sample (n=193,617 person-months) from the Army STARRS Historical Administrative Data Study (HADS). Control person-months were assigned a weight of 200 to adjust for under-sampling.

[2]Total includes both cases (i.e., soldiers with a suicide attempt) and control person-months.

[3]Rate per 100,000 person-years, calculated based on n_1/n_2, where n_1 is the unique number of soldiers within each category and n_2 is the annual number of person-*years*, not person-*months*, in the population (n=3.08 million).

[4]Pop % = percent of the population of enlisted soldier.

[5]SRE = Standardized risk estimates (suicide attempters per 100,000 person-years) were calculated assuming other predictors were at their sample-wide means. SREs were calculated based on logistic regression models that included gender, age at entry into the Army, current age, race/ethnicity, education, marital status, time in service (≤ 1 year, 2 years, 3-4 years, 5-10 years, >10 years), deployment status (never, currently, or previously deployed), and military occupation. The models also included a dummy predictor variable for calendar month and year to control for secular trends.

Table S9a. Pairwise tests examining multivariate associations of time in service with suicide attempt among Regular Army enlisted soldiers stratified by military occupation.[1,2]

Time in service	Occupation					
	Combat arms		Combat medic		Other	
	OR	(95% CI)	OR	(95% CI)	OR	(95% CI)
≤ 1 year vs. 2 years	1.0	(0.9–1.2)	1.4*	(1.1–1.7)	1.3*	(1.2–1.4)
≤ 1 year vs. 3-4 years	1.3*	(1.1–1.5)	2.1*	(1.6–2.8)	1.9*	(1.7–2.0)
≤ 1 year vs. 5-10 years	1.8*	(1.5–2.3)	3.3*	(2.2–4.8)	3.2*	(2.8–3.6)
≤ 1 year vs. >10 years	5.9*	(4.0–8.7)	5.9*	(3.1–11.1)	6.9*	(5.6–8.5)
2 years vs. 3-4 years	1.2*	(1.1–1.4)	1.5*	(1.2–2.0)	1.5*	(1.4–1.6)
2 years vs. 5-10 years	1.8*	(1.5–2.1)	2.4*	(1.6–3.4)	2.5*	(2.2–2.8)
2 years vs. >10 years	5.7*	(3.9–8.2)	4.2*	(2.3–7.8)	5.4*	(4.4–6.6)
3-4 years vs. 5-10 years	1.4*	(1.2–1.7)	1.6*	(1.1–2.1)	1.7*	(1.5–1.9)
3-4 years vs. ≥10 years	4.6*	(3.2–6.5)	2.8*	(1.6–4.9)	3.7*	(3.1–4.4)
5-10 years vs. >10 years	3.2*	(2.3–4.4)	1.8*	(1.1–3.0)	2.2*	(1.8–2.6)
χ^2_4	88.8*		45.4*		448.4*	

Table S9b. Pairwise tests examining multivariate associations of military occupation with suicide attempt among Regular Army enlisted soldiers stratified by time in service.[1,2]

Occupation	Time in service									
	≤ 1 year		2 years		3-4 years		5-10 years		>10 years	
	OR	(95% CI)	OR	(95% CI)	OR	(95% CI)	OR	(95% CI)	OR	(95% CI)
Combat arms vs. other	1.1	(1.0–1.2)	1.2*	(1.0–1.3)	1.2*	(1.1–1.3)	1.4*	(1.2–1.5)	1.1	(0.9–1.4)
Combat medic vs. other	1.5*	(1.3–1.7)	1.4*	(1.2–1.7)	1.3*	(1.1–1.6)	1.2	(1.0–1.5)	1.4	(1.0–2.1)
Combat medic vs. combat arms	1.4*	(1.2–1.6)	1.2	(1.0–1.5)	1.1	(0.9–1.3)	0.9	(0.7–1.2)	1.3	(0.8–2.0)
χ^2_2	44.9*		19.1*		18.7*		25.4*		3.7	

[1]The sample of enlisted soldiers (n=9,650 cases, 153,523 control person-months) is a subset of the total sample (n=193,617 person-months) from the Army STARRS Historical Administrative Data Study (HADS). Control person-months were assigned a weight of 200 to adjust for under-sampling.

[2]Logistic regression models included gender, age at Army entry, current age, race/ethnicity, education, marital status, time in service (≤ 1 year, 2 years, 3-4 years, 5-10 years, >10 years), deployment status (never, currently, or previously deployed), and military occupation. The models also included a dummy predictor variable for calendar month and year to control for secular trends.

*$p < 0.05$

Table S10. Counts and rates of suicide attempts by socio-demographic and service-related variables among Regular Army enlisted soldiers stratified by military occupation.[1]

	Occupation														
	Combat arms (n= 38,289)					Combat medic (n= 8,033)					Other (n= 114,995)				
	Cases (n)	Total (n)[2]	Rate[3]	Pop %[4]	SRE[5]	Cases (n)	Total (n)[2]	Rate[3]	Pop %[4]	SRE[5]	Cases (n)	Total (n)[2]	Rate[3]	Pop %[4]	SRE[5]
**Gender**															
Male	2,506	7,159,106	420	100.0	420	383	1,115,783	412	75.9	423	4,309	17,864,909	289	82.3	289
Female	–	–	–	–	–	299	355,099	1,010	24.1	935	2,137	3,851,337	666	17.7	664
**Age at army entry**															
< 21	1,767	4,726,967	449	66	424	414	810,214	613	55.1	568	4,281	13,341,081	385	61.4	366
21–24	539	1,722,739	375	24.1	422	164	396,164	497	26.9	516	1,430	5,302,430	324	24.4	340
25+	200	709,400	338	9.9	383	104	264,504	472	18.0	580	735	3,072,735	287	14.1	337
**Current age**															
< 21	910	1,371,310	796	19.2	560	245	182,845	1,608	12.4	786	2,158	3,058,558	847	14.1	425
21–24	952	2,508,752	455	35	393	222	401,222	664	27.3	535	2,321	6,260,121	445	28.8	356
25–29	428	1,577,028	326	22	337	121	376,721	385	25.6	433	1,204	5,080,004	284	23.4	321
30–34	139	806,339	207	11.3	352	65	227,465	343	15.5	555	428	3,131,628	164	14.4	288
35–39	53	591,053	108	8.3	286	16	182,416	105	12.4	226	229	2,499,429	110	11.5	275
40+	24	304,624	95	4.3	337	13	100,213	156	6.8	413	106	1,686,506	75	7.8	250
**Race/ethnicity**															
White	2,027	5,200,627	468	72.6	439	513	899,113	685	61.1	606	4,255	11,955,655	427	55.1	404
Black	154	780,154	237	10.9	355	76	296,476	308	20.2	449	1,184	5,875,584	242	27.1	278
Hispanic	225	781,225	346	10.9	350	55	173,655	380	11.8	403	699	2,574,699	326	11.9	303
Asian	53	258,853	246	3.6	285	30	71,430	504	4.9	539	202	878,402	276	4.0	274
Other	47	138,247	408	1.9	540	8	30,208	318	2.1	451	106	431,906	295	2.0	385
**Education**															
< High school[6]	918	1,273,918	865	17.8	668	182	143,982	1,517	9.8	1,054	1,787	2,445,987	877	11.3	605
High school	1,542	5,481,142	338	76.6	353	449	1,122,649	480	76.3	478	4,375	16,615,575	316	76.5	318
Some college	26	215,226	145	3.0	250	23	109,023	253	7.4	413	142	1,353,342	126	6.2	232
≥ College	20	188,820	127	2.6	173	28	95,228	353	6.5	479	142	1,301,342	131	6.0	181
**Marital status**															
Never married	1,425	3,277,825	522	45.8	393	426	602,026	849	40.9	576	3,584	8,609,584	500	39.6	342
Currently married	1,044	3,683,644	340	51.5	463	241	795,241	364	54.1	533	2,680	12,088,680	266	55.7	375

Previously married	37	197,637	225	2.8	440	15	73,615	245	5.0	446	182	1,017,982	215	4.7	394
**Time in service**															
≤ 1 year	800	1,211,400	793	16.9	578	301	230,101	1,570	15.6	998	2,297	3,039,497	907	14.0	668
2 years	545	1,136,945	575	15.9	558	133	186,533	856	12.7	717	1,334	2,708,534	591	12.5	527
3–4 years	673	1,847,073	437	25.8	450	131	323,531	486	22.0	474	1,473	4,607,273	384	21.2	358
5–10 years	409	1,720,809	285	24.0	313	87	394,287	265	26.8	305	1,043	6,118,843	205	28.2	211
> 10 years	79	1,242,879	76	17.4	98	30	336,430	107	22.9	170	299	5,242,099	68	24.1	97
**Deployment status**															
Never deployed	1,286	2,374,686	650	33.2	498	480	667,680	863	45.4	579	4,117	9,254,117	534	42.6	377
Currently deployed	262	2,053,262	153	28.7	152	59	304,259	233	20.7	302	619	4,747,419	157	21.9	181
Previously deployed	958	2,731,158	421	38.1	576	143	498,943	344	33.9	714	1,710	7,714,710	266	35.5	454

[1]The sample of enlisted soldiers (n=9,650 cases, 153,523 control person-months) is a subset of the total sample (n=193,617 person-months) from the Army STARRS Historical Administrative Data Study (HADS). Control person-months were assigned a weight of 200 to adjust for under-sampling.

[2]Total includes both cases (i.e., soldiers with a suicide attempt) and control person-months.

[3]Rate per 100,000 person-years, calculated based on n_1/n_2, where n_1 is the unique number of soldiers within each category and n_2 is the annual number of person-*years*, not person-*months*, in the population (n=3.08 million).

[4]Pop % = percent of the population of enlisted soldier.

[5]SRE = Standardized risk estimates (suicide attempters per 100,000 person-years) were calculated assuming other predictors were at their sample-wide means. SREs were calculated based on logistic regression models that included gender, age at entry into the Army, current age, race/ethnicity, education, marital status, time in service (≤ 1 year, 2 years, 3-4 years, 5-10 years, >10 years), deployment status (never, currently, or previously deployed), and military occupation, The models also included a dummy predictor variable for calendar month and year to control for secular trends.

[6]< High School includes: General Educational Development credential (GED), home study diploma, occupational program certificate, correspondence school diploma, high school certificate of attendance, adult education diploma, and other non-traditional high school credentials.
